# MimoDB: a New Repository for Mimotope Data Derived from Phage Display Technology

**DOI:** 10.3390/molecules15118279

**Published:** 2010-11-15

**Authors:** Beibei Ru, Jian Huang, Ping Dai, Shiyong Li, Zhongkui Xia, Hui Ding, Hao Lin, Feng-Biao Guo, Xianlong Wang

**Affiliations:** Key Laboratory for Neuroinformation of Ministry of Education, School of Life Science and Technology, University of Electronic Science and Technology of China, Chengdu, Sichuan 610054, China; Email: rubeibei1988@gmail.com (B.R.); pingdai@263.net (P.D.); lishiyonglee@163.com (S.L.); xiazhk@163.com (Z.X.); hding@uestc.edu.cn (H.D.); hlin@uestc.edu.cn (H.L.); fbguo@uestc.edu.cn (F.G.); chem.wang@gmail.com (X.W.)

**Keywords:** mimotope, phage display, biopanning, combinatorial libraries, target-unrelated peptides

## Abstract

Peptides selected from phage-displayed random peptide libraries are valuable in two aspects. On one hand, these peptides are candidates for new diagnostics, therapeutics and vaccines. On the other hand, they can be used to predict the networks or sites of protein-protein interactions. MimoDB, a new repository for these peptides, was developed, in which 10,716 peptides collected from 571 publications were grouped into 1,229 sets. Besides peptide sequences, other important information, such as the target, template, library and complex structure, was also included. MimoDB can be browsed and searched through a user-friendly web interface. For computational biologists, MimoDB can be used to derive customized data sets and benchmarks, which are useful for new algorithm development and tool evaluation. For experimental biologists, their results can be searched against the MimoDB database to exclude possible target-unrelated peptides. The MimoDB database is freely accessible at http://immunet.cn/mimodb/.

## 1. Introduction

Functional peptides can be selected from the phage-displayed random peptide libraries with various substances ranging from small compounds to whole organisms. The substance used to screen the phage display library is usually called the target; the natural partner binding to the target is named the template. Peptides mimicking the binding site on the template and binding to the target are called mimotopes. Since its introduction 25 years ago, phage display technology has been widely adopted as a tool for studying the sites and the networks of protein-protein interactions [1,2,3,4,5]. It has also shown promise for the generation of diagnostics, therapeutics and vaccines [6,7,8,9,10,11,12]. All these studies and achievements, whether basic or applied, computational or experimental, are based on the mimotopes acquired from the phage display experiments. Collecting information on mimotopes is thus of great importance.

Like other types of sequence data, the amount of peptide sequence data derived from phage display has been accumulating rapidly. The number and diversity of phage display experiments has risen to the point where it is now essential to gather all the sequence data and other associate information into a comprehensive, continuously updated database. Fortunately, there is a database for mimotopes called ASPD which has 195 sets of mimotopes from 112 papers [13]. But, as far as we know, it has not been updated since August 2001.

In this paper, we describe the MimoDB database, a new repository for mimotope data derived from phage display technology. This database complements the ASPD and other related databases.

## 2. Results and Discussion

### 2.1. Database Content

In the current release, 10,716 peptides grouped into 1,229 sets are collected from 571 published papers. These peptides are selected with 775 different targets from 250 phage libraries. The type of targets is quite diverse, varying from small compounds to nucleic acids, proteins, cells, tissues, organs and even entire organisms. Nonetheless, proteins including antibodies and receptors from human and mouse are the most used targets. At present, there are 257 known templates in MimoDB. For most of the peptides, their templates are not determined. The database also stores 53 solved structures for target-template complex, which are related to 63 mimotope sets. There are five solved structures for target-mimotope complex, which are related to four mimotope sets. For more information on the statistics of the database content, please visit the statistics page of the MimoDB database.

### 2.2. User Interface

**Figure 1:**
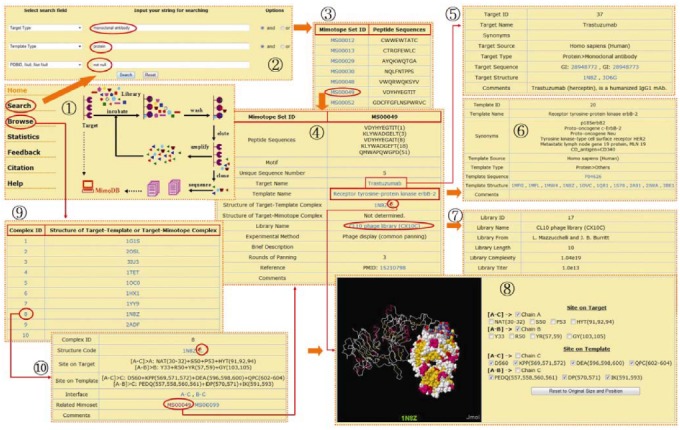
A series of screenshots showing navigation in the MimoDB database. **(1) **Homepage of MimoDB. **(2)** Search interface. **(3)** Search results. **(4)** Browse an entry of mimotope set. **(5)** Browse the related target. **(6)** Browse the related template. **(7)** Browse the related library. **(8)** View the interaction sites between target and template interactively. **(9)** Summary table of complex structure. **(10)** Browse an entry of target-template complex structure.

Users can access the MimoDB database freely at URL http://immunet.cn/mimodb, where first a welcome page will be shown with an animated image depicting the phage display technology [see screenshot (1) in Figure 1]. 

Clicking the “Search” menu item on the left side of the home page opens the search interface. MimoDB can be searched by most fields in the five main tables and by any combination of up to three of the fields. There are three pull-down list boxes and two pairs of radio buttons on the search interface. A query is formed by selecting one or more fields from the pull-down list box and combining them logically by "and" or "or" radio buttons. Any words, strings and numbers such as peptide sequence, protein name, species name, PDB code, PMID of reference, can be entered into the blank text forms. For the option “PDBID, Null, Not Null” of the target-template complex option group in the list box, two special words, *i.e.* “null” and “not null” are supported, which mean with or without solved structure of target-template complex. After the search button is pressed, the search form will parse the criteria into SQL database queries. The result returned is formatted into a paged summary table. Only the mimotope set ID and the first sequence in the mimotope set are shown. Selecting each ID in the summary table opens a new window that displays the detailed information of each mimotope set.

For example, if one wanted to retrieve all mimotope sets selected with monoclonal antibodies against protein antigens and at the same time the structures of corresponding antibody-antigen complexes have been solved, the user would first select the search field "Target type" from the pull-down list box, input "monoclonal antibody" into the corresponding blank text form and then select the "and" button. Then the user would select the "Template Type" search field, input "protein" and select the "and" button. Finally, the user would select "PDBID, Null, and Not Null" field from the target-template complex option group and input "not null". The operations above will make the search interface appear as the screenshot (2) in Figure 1. After clicking on the "Search" button, records fulfilling the requirements would appear in a new window as the screenshot (3) in Figure 1. Following the hyperlinks in the mimotope set table, other background information on target, template and library can be found [see screenshots (4)-(7) in Figure 1]. 

Users can access the detailed information on a mimotope set not only from search result but also from the homepage of MimoDB. Click the “Browse” menu item on the left side of the home page, the browse interface will appear. As the mimotope set table is most important and experiment-dependent, the browsing starts from the mimotope set table by default. As described previously, a paged summary table will appear at first. Users can browse the five main tables (*i.e.* mimotope set, target, template, library and structure) by clicking the corresponding menu item on the left side of browse interface [see screenshot (9) and (10) in Figure 1].

The interaction sites between the target and template or between target and mimotope can be viewed interactively in the context of corresponding 3D structure. The view interface is created by JmolApplet scripts and PHP codes. When browsing or searching the MimoDB database, mimotope set entry with solved structures of target-template or target-mimotope complex will have a conspicuous view icon beside its PDB code. Clicking the view icon will initialize the loading of the JmolApplet. By default, the complex structure is displayed as backbone colored by secondary structure. After loading, users can turn on or turn off the residues and segments composing the interaction sites, and the chains of target, templates or mimotopes. When turned on, residues and segments "blink" and are then displayed in spacefill mode colored by CPK [see screenshot (8) in Figure 1]. Users can also zoom in, zoom out, move, spin, and rotate the structure, or even measure distances, angles, and dihedral angles. A help page for viewing can be found by clicking the “help” menu item on the left side of the view interface.

We have tested the user interface of the MimoDB database with the Internet Explorer, Mozilla Firefox, Google Chrome, Opera and Safari on Windows, Linux or Mac OS platforms. Although it looks a little bit different among different browsers on different platforms, it works normally on all tested browsers and platforms.

### 2.3. Database Usage

To predict the protein interaction site based on mimotopes is a fascinating work for computational biologists. Quite a few methods and tools such as SiteLight, 3DEX, MIMOP, MIMOX, Mapitope, Pepsurf, Pepitope, Pep-3D-Search, and Episearch *etc*. have been developed in recent years [14,15,16,17,18,19,20,21,22]. Although these tools have succeeded in some cases, none of them has been evaluated systematically due to the lack of data resources and benchmarks. With the MimoDB database, it is convenient now for the community to produce benchmarks and customized data sets via the search interface. As shown previously, researchers can easily get 18 sets of mimotopes with solved structures of corresponding antibody-antigen complex. This data set will be quite useful for the study of mimotope-based epitope mapping. Similarly, there are 18 sets of mimotopes with solved structures of corresponding receptor-ligand complex and 27 sets of mimotopes for other protein-protein interactions with complex structure solved. In total, it makes a benchmark with 63 sets of data for mimotope-based protein-protein interaction study. The MimoDB database is not only useful for benchmarking the existing tools, but also helpful for developing new and better algorithm to predict protein-protein interaction sites based on mimotopes.

Experimental biologists are frequently annoyed by target-unrelated peptides [23,24]. These peptides are displayed on phage, reacting with contaminants or other components of the screening system rather than the actual target site. From time to time, target-unrelated peptides are recovered with those target-specific binders (mimotopes), making the experiment results confusing. We have developed a tool called SAROTUP to scan and filter target-unrelated peptides with known motifs [25]. SAROTUP can improve the performance of computational tools for epitope prediction based on phage display. However, a lot of target-unrelated peptides bear no known motifs. The coming of MimoDB makes it possible now to find out target-unrelated peptides bearing no known motifs through database search. Experimental biologists can search the MimoDB database to find out if their results have appeared in the database. If so, the match might mean that different research groups have isolated the same peptide with different targets. In this situation, the peptide may not be a true target binder. 

Furthermore, the large amount of sequences in the MimoDB database makes it feasible to find new target-unrelated peptides and new patterns for target-unrelated peptides. In our preliminary analysis, among the 10,716 peptides in MimoDB, 9,802 peptides appear only once; there are 378 peptides that appear 914 times. The most frequent peptides are SVSVGMKPSPRP, HAIYPRH and LPLTPLP, which are seen in 22, 11 and 10 sets of mimotopes selected with different targets. The magic peptide SVSVGMKPSPRP has been doubted to be a target-unrelated peptide [26,27]; HAIYPRH has been proved to be a target-unrelated peptide [24]; to our knowledge, this is the first time that LPLTPLP is doubted to be a new target-unrelated peptide.

### 2.4. Future Development

The MimoDB database will be revised and updated quarterly. Errors occurred during the data accumulation phase will be corrected; mimotope sets and related information in newly published peer-review articles will be added. It can be expected that both the quantity and quality of MimoDB records will be improved. To make the MimoDB database more comprehensive, we will search patent databases and other literature databases in future to increase its data coverage. Furthermore, an online submission system is also under consideration. In addition, we are going to include peptides selected from random peptide library not only through phage display experiments, but also other similar surface display technology such as ribosome display, yeast display and bacterial display. 

## 3. Materials and Methods

### 3.1. Data Collection and Organization

Query was made with the unquoted word “phage display” against all fields of PubMed indexed literatures published before March 31, 2010. The search returned 4,568 records. Each record was then screened manually. Only the papers with peptide selected from phage-displayed random peptide libraries were kept. Those papers on phage-displayed cDNA libraries, including antibody phage display libraries, were dropped. The papers without sequence data or peptide length longer than 40 amino acids were also excluded. At last, peptide sequences and other related information were manually extracted from 571 papers with available full text. 

The sequence data is organized as mimotope sets rather than individual sequence. In most cases, peptides are grouped into a mimotope set if they are from the same independent experiment with identical experimental method, condition and parameters. If available, the appearing times (an integer) or frequency (a decimal between zero and one) of selected peptides are collected and put in the brackets right behind each sequence. While such experiment-dependent data are sourced from the published paper directly, other data such as background information for target, template and structure, which are quite independent from the phage display experiments, are mainly extracted from the external databases such as Uniprot, GenBank and PDBsum [28,29,30].

### 3.2. Database Design and Implementation

**Figure 2:**
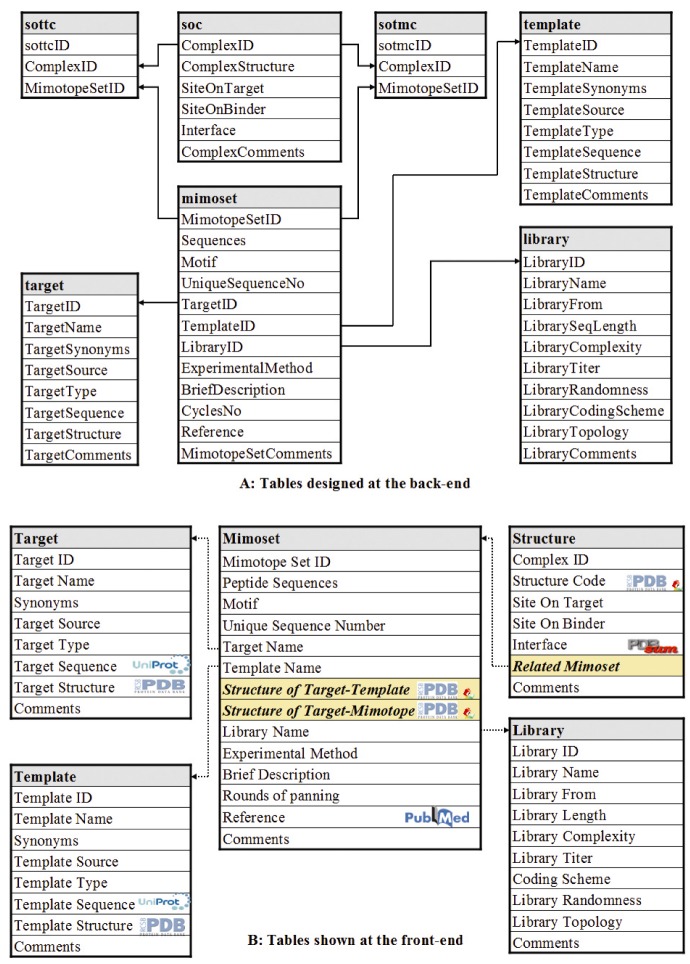
Infrastructure of the MimoDB Database. **(A)** Tables designed at the back-end. **(B) **Tables shown at the front-end. The relations among tables at the back-end are shown with solid arrow. Tables shown to users at the front-end are generated on the fly based on the tables and their relations at the back-end. The dotted arrow lines indicate that the content is linked internally to other tables. The logo in a cell means that the content in that cell is hyperlinked to the corresponding external database except the small view icon in the field of Structure Code, which is linked to a molecular visualization tool provided by MimoDB. The cells with background color in sallow indicate fields not existed in the tables at the back-end but added to the table at the front-end dynamically.

The MySQL relational database management system is used to store and manage the data. At the back-end, five main tables and two joint tables are created. Most table names and field names are self-evident and the meaning of each table and each field in detail are given on the help page of MimoDB. The relationships among the tables are shown in Figure 2. The core table is named as “mimoset”, which stores mimotope set and the related information such as the target used, the template mimicked, the complex structure indentified, the reference, the experiment method and condition, *etc*. The MimoDB database is designed to hyperlink several mainstream external databases such as Uniprot, GenBank, PDB and PDBsum, providing wide background information for each mimotope set.

The web interface is coded in PHP with PEAR database abstraction layer support. At the front-end, the content of the MimoDB database are shown in five different tables corresponding to the five main tables at the back-end (see Figure 2). The tables shown at the front-end are generated to be more user-friendly. For example, target ID, template ID and library ID are replaced with corresponding names. Powered by the two joint tables not shown at the front-end, two additional fields, *i.e.* the structure of target-template complex and the structure of target-mimotope complex, are added to the mimotope set table dynamically. This is also the case for the table of the structure of complex, where mimotope sets related to a complex structure are added on-the-fly. For structures of target-template or target-mimotope complex, an interactive view function is implemented with JmolApplet.

## 4. Conclusions

Mimotopes acquired from phage display technology are of great importance in basic and applied research. Here we described a new mimotope database called MimoDB, which has 10,716 peptides grouped into 1,229 sets at present. This database can be used by computational biologists to derive customized data sets and benchmarks, which are useful for new algorithm development and tool evaluation. It also can be used by experimental biologists to exclude possible target-unrelated peptides through searching the database. MimoDB is freely available at http://immunet.cn/mimodb.
